# Genome‐wide characterization and function analysis uncovered roles of wheat LIMs in responding to adverse stresses and *TaLIM8‐4D* function as a susceptible gene

**DOI:** 10.1002/tpg2.20246

**Published:** 2022-07-27

**Authors:** Yiting Li, Xi Liu, Yongxin Xiao, Yong Wen, Keke Li, Zhaolan Ma, Lijun Yang, Yongxing Zhu, Junliang Yin

**Affiliations:** ^1^ Hubei Key Laboratory of Waterlogging Disaster and Agricultural Use of Wetland/College of Agriculture/College of Horticulture and Gardening Yangtze Univ. Jingzhou Hubei 434025 China; ^2^ Key Laboratory of Integrated Pest Management of Crops in Central China, Ministry of Agriculture/Hubei Key Laboratory of Crop Diseases, Insect Pests and Weeds Control, Institute of Plant Protection and Soil Science Hubei Academy of Agricultural Sciences Wuhan Hubei 430064 China

## Abstract

The Lin‐11, Isl‐1, and Mec‐3 domains (LIM) transcription factors play essential roles in regulating plant biological processes. Despite that, there is a lack of a full understanding of LIMs in wheat (*Triticum aestivum* L.). In this study, 28 wheat LIM s (TaLIMs) were identified and designated as TaLIM1‐1A to TaLIM12‐7D. The *cis*‐regulatory element analysis showed that TaLIMs were rich in elements related to biological and abiotic stresses. Expression profiling analysis showed that certain members of TaLIMs were responsive to biotic and abiotic stresses, such as *TaLIM1‐1A*, *TaLIM3‐2B*, *TaLIM8‐4D*, and *TaLIM10‐5D*, were significantly induced by heat, drought, sodium chloride (NaCl), abscisic acid (ABA) and *Fusarium graminearum* stresses. Furthermore, the biological function of *TaLIM8‐4D* was analyzed and results showed that it was subcellular localization in the nucleus and could induce weak cell death in *Nicotiana benthamiana* leaves. Additionally, overexpression of *TaLIM8‐4D* could upregulate plant *pathogenesis‐related* (*PR*) genes, promoting the infection of hemibiotrophic pathogen, implying that *TaLIM8‐4D* could function as susceptible gene in the nucleus by upregulating *PR* genes and inducing cell death to promote the colonization of hemibiotrophic agent *F*. *graminearum*. Overall, the systematic identification, characterization, expression profiling, evolutionary, and function analyses provided the ability to understand TaLIMs and laid a foundation for the further function study of LIM family members in wheat.

## Core Ideas

- The wheat genome contains 28 *TaLIM* genes.
- *TaLIMs* are responsive to abiotic and biotic stresses.
- *TaLIM8‐4D* is a cell‐death inducer localized in the nucleus.
- *TaLIM8‐4D* was a susceptible gene and its overexpression can promote the infection of *Fusarium graminearum*.
- *TaLIM8‐4D* upregulates *PR* genes and induces cell death to promote the colonization of hemibiotrophic pathogen.

**Abbreviations**
aa: amino acid
ABA: abscisic acid
ARE: anaerobic induction
AtLIM: *Arabidopsis thaliana* LIM
cDNA: complementary DNA
Ka: nonsynonymous substitution rate
Ks: synonymous substitution rate
LIM: Lin‐11, Isl‐1, and Mec‐3 domains
OsLIM: *Oryza sativa* LIM
qRT‐PCR: quantitative real‐time polymerase chain reaction
TaLIM: *Triticum aestivum* LIM
ZmLIM: *Zea mays* LIM

## 1 INTRODUCTION

Transcription factors, also known as *trans*‐acting factors, are a kind of protein that can regulate the expression of target genes (Yin et al., 2020). They participate in the regulation of plants’ response to environmental stresses by directly or indirectly binding to *cis*‐elements in the promoter region of stress‐induced genes (Chen et al., 2017). Typical transcription factors contain several special structures that determine the transcription and regulation characters of corresponding transcription factors, such as DNA binding domain, activation domain, oligomerization site, and nuclear localization signal (Eliasson et al., 2000; Fang, Jiang et al., 2020; Liu et al., 2001). Lin‐11, Isl‐1, and Mec‐3 domains (LIM) proteins are widely present in eukaryotes. They are a class of cysteine‐rich proteins and comprise a key transcription factor family (Way & Chalfie, 1988). Sequence analysis showed that all LIM proteins contain at least one LIM domain, which is a cystine‐ and histidine‐rich zinc coordination domain and functionally related to DNA binding during the transcription process (Dawid et al., 1998). The LIM domain sequence [(C–X_2_–C–X_16_–_23_–H–X_2_–C) –X_2_– (CX_16_–_23_–Z–X_2_–C)] is highly conserved in many species and generally comprised of >50 amino acid residues (Dawid et al., 1995).

Previous studies showed that LIM proteins mainly have two functions in plants: (a) as a transcription factor, it regulates the expression of genes related to phenylpropanoid biosynthesis pathway, thus it is involved in plant secondary cell wall development and cell differentiation (Kawaoka & Ebinuma, 2001); (b) as a binding protein of actin, it regulates the cytoskeleton structure of actin, therefore it participates in the cytoskeleton organization (Maul et al., 2003). Considering their important roles, LIM family members had been studied in a variety of plants such as black cottonwood (*Populus trichocarpa* Torr. & A. Gray ex Hook.), *Arabidopsis thaliana* (L.) Heynh., rice (*Oryza sativa* L.) (Arnaud et al., 2007), maize (*Zea mays* L.) (Zhang et al., 2013), tomato (*Solanum lycopersicum* L.) (Khadiza et al., 2016), and quinoa (*Chenopodium quinoa* Willd.) (Zhu, Wang et al., 2021). However, there is no comprehensive study of LIM family in wheat (*Triticum aestivum* L.).

As a grain plant in Gramineae, wheat is widely grown throughout the world; therefore, it is one of the most important food crops worldwide (Zhou et al., 2020). However, the growth of wheat is seriously threatened by many abiotic and biotic stresses (Fang, Jiang et al., 2020; Zhu, Gong, & Yin, 2019). Breeding stress‐tolerant cultivars is the most economic and effective way to reduce the loss caused by abiotic and biotic stresses. Since resistance genes are the foundation for breeding stress‐tolerant cultivars, it is necessary to mine more resistant genes (Zhu, Jia et al., 2019). In this study, the wheat LIM (TaLIM) family members were systemically identified and characterized by bioinformatics method. Their chromosome distribution, gene structure, motif, *cis*‐acting elements, expression patterns, and subcellular localization were further analyzed. Furthermore, the biological function of *TaLIM8‐4D* was analyzed. These results can provide a theoretical basis for the further study of LIM gene function in wheat and prepare gene resources for resistance breeding.

**Core Ideas**

- The wheat genome contains 28 *TaLIM* genes.
- *TaLIMs* are responsive to abiotic and biotic stresses.
- *TaLIM8‐4D* is a cell‐death inducer localized in the nucleus.
- *TaLIM8‐4D* was a susceptible gene and its overexpression can promote the infection of *Fusarium graminearum*.
- *TaLIM8‐4D* upregulates *PR* genes and induces cell death to promote the colonization of hemibiotrophic pathogen.

## 2 MATERIALS AND METHODS

### 2.1 Identification of LIM gene family members from the wheat genome

The wheat reference genome (IWGSC v2.1) was downloaded from Wheat Whole Genome Database (https://wheat‐urgi.versailles.inra.fr/Seq‐Repository/Assemblies). The hidden Markov model of LIM domain (PF00412) was downloaded from Pfam (v32.0, http://pfam.xfam.org/) and used as query sequences to perform the first‐round BLASTp against wheat reference protein sequences (e‐value < 10^−10^). Meanwhile, 34 reported LIM protein sequences, including 14 LIMs from *Arabidopsis* (AtLIMs, TAIR10, http://www.arabidopsis.org/index.jsp), nine LIMs from rice (OsLIMs, RGAP, http://rice.plantbiology.msu.edu/index.shtml) (Arnaud et al., 2007), and 11 LIMs from maize (ZmLIMs, maizeGDB, https://www.maizegdb.org/) (Zhang et al., 2013), were collected and used as query sequences to perform the second‐round BLASTp. After merging two rounds of BLASTp search results and deleting redundant sequences, unique ones were further validated through Pfam (v32.0, http://pfam.xfam.org/) and InterProScan (v71.0, http://www.ebi.ac.uk/InterProScan) to determine whether sequences contain PF00412 domain and exclude sequences that do not contain LIM domain.

### 2.2 Phylogenetic analysis of wheat LIMs

Multiple sequence alignments of the identified LIM protein sequences were performed using the ClustalW2 program through the neighbor‐joining method with 1,000 replicated bootstraps (Thompson et al., 1994). A phylogenetic tree, containing TaLIMs, AtLIMs, OsLIMs, and ZmLIMs, was further illustrated using ITOL tools (Interactive Tree of Life, http://itol.embl.de) to infer the phylogenetic relationships of TaLIMs and assisting in the naming of them.

### 2.3 Chromosomal mapping, gene duplication and Ka/Ks analysis

Detailed information of corresponding TaLIMs were extracted from GFF3 file. The software MapInspect was used to mark the start and end positions of TaLIMs to each chromosome (Song et al., 2019). Gene duplicates are divided into tandem duplicates and fragment duplicates. Tandem repeated events were identified using the following assessment criteria: (a) aligned sequence length >80% regions of each sequence, (b) identity >80%, (c) threshold ≤10^−10^, (d) only one duplication can be admitted when genes are linked closely, and (e) intergenic distance is <25 kb. If genes satisfied criteria (a), (b), and (c) and located on different chromosomes, they were judged as segmental duplications (He et al., 2020). The software TBtools was used to calculate the nonsynonymous substitution rate (Ka) and synonymous substitution rate (Ks) of TaLIM duplication pairs (Chen et al., 2020).

### 2.4 Gene structure, motif, and *cis*‐element analysis of TaLIMs

According to GFF3 annotation, the gene exon–intron structures of *TaLIMs* were drawn by TBtools. The TaLIMs protein structures were analyzed by Pfam, and results were used to visualize protein functional domains (Biological Sequence, http://ibs.biocuckoo.org/) (Liu et al., 2015). The online tool MEME (v5.3.1, http://meme‐suite.org/tools/meme) was used to identify the conserved motifs from TaLIMs according to the following conditions. Each sequence could contain any number of nonoverlapping motifs, the number of different motifs was 15, and the width of motifs ranged from 6 to 50 aa (amino acid). The output results were explored by TBtools to draw the motif structures of TaLIM proteins. Function of these motifs were further predicted using the online tool in Pfam. The upstream promoter sequences (1–1,500 bp) were extracted manually from genome sequence and uploaded into PlantCARE (http://bioinformatics.psb.ugent.be/webtools/plantcare/html/) to identify the *cis*‐elements in promoter regions. Then R package pheatmap was used to sort out and display the analysis results (Huang et al., 2021).

### 2.5 Protein characterization and homology modeling of TaLIMs

The protein basic characteristics of the TaLIMs, including the length of the protein, molecular weight, isoelectric point, grand average of hydropathicity, were analyzed using ExPASy Server10 (https://prosite.expasy.org/PS50011). Signal peptide was predicted with SignalP 5.0 (http://www.cbs.dtu.dk/services/SignalP/), and subcellular localization was predicted with Plant‐mPLoc (http://www.csbio.sjtu.edu.cn/bioinf/plant‐multi/?tdsourcetag=s_pcqq_aiomsg). The three‐dimensional homology modeling of TaLIMs were performed using online server SWISS‐MODEL (https://www.swissmodel.expasy.org/).

### 2.6 Expression pattern profiling of *TaLIM* genes

Wheat transcriptome sequencing data were downloaded from the NCBI Short Read Archive database and aligned to the wheat reference genome using Hisat2 (Fang, He et al., 2020). Then, the expression levels of *TaLIMs* genes were calculated and normalized by Cufflinks (represented by TPM, transcripts per kilobase of exon model per million mapped reads) (Trapnell et al., 2012). Log_2_(TPM + 1) values were used to draw heatmap by R package ‘pheatmap’ to show the expression pattern of *TaLIM* genes under different conditions (Jiang et al., 2021; Zhu et al., 2020).

### 2.7 Interspecies evolution analysis of *TaLIM* genes

The homologous of *LIM* genes in red wild einkorn (*T. urartu* Tumanian ex Gandilyan), wild emmer [*T. turgidum* L. subsp. *dicoccoides* (Körn. ex Asch. & Graebn.) Thell.], and rough‐spike hard grass (*Aegilops tauschii* Coss.) were identified by reciprocal BLASTp analysis (Jiang et al., 2020). The cutoff values (e‐value < 10^−10^, identity > 80%) were used to ensure the reliability of homologous. The R package ‘circlize’ was used to indicate the chromosomal relationship between them (Song et al., 2019).

### 2.8 Growth and stress treatment of wheat seedlings

Seeds of common hexaploid wheat cultivar Yangmai 20, which is moderatly susceptible to powdery mildew and *Fusarium* head blight, were surface sterilized with 1% H_2_O_2_, rinsed thoroughly with distilled water, and germinated in petri dishes above three layers of filter paper saturated with water at 25 °C for 2 d (Zhang et al., 2019). Seedlings were then transferred and cultured in one‐quarter strength Hoagland solution and increased to one‐half strength after 3 d (Zhang et al., 2019). *Fusarium graminearum* (PH‐1) was routinely cultured on potato dextrose agar plate and then cultured in liquid mung bean medium (60 g L^−1^) with 25 °C and 150 rpm to produce spores. When coleoptiles reached 0.5 cm, seedlings were soaked in spore suspension (5 × 10^5^ spores ml^−1^) for 1 min (Yang et al., 2010) then wrapped in a piece of moist paper towel and cultured at 25 °C with relative humidity 65%. Seedlings coleoptiles were sampled at 1, 2, 3, and 5 d after inoculation. When seedlings grew into one leaf and one heart stage, 150 mM NaCl was used to treat plants. The pH of the nutrient solution was adjusted to 6.0 every 2 d using 1 M KOH or 0.2 M H_2_SO_4_. Growth conditions are 16 h/8 h (day/night) photoperiod at 25 and 20 °C, respectively. Leaves were collected at 2, 6, 12, 24, and 48 h after treatment. For ABA treatment, one‐half strength Hoagland solution containing 100 μM ABA was used to culture the wheat seedlings under conditions as mentioned above (Song & Peng, 2019). After 2, 6, 12, 24, and 48 h, leaf samples were harvested. For heat and drought treatment, germinated seeds were planted in a mix of peat moss and vermiculite (v/v = 1:1) and cultured under 25 °C day and 23 °C night with 16 and 8 h light vs. dark photoperiod until flowering stage. Then one group of plants were placed in a light incubator under heat at 16 and 8 h light vs. dark photoperiod. Another group was irrigated with water containing 20% PEG6000 to induce drought treatment (Gao, 2016). The pollen tissues were harvested after 12, 24, 48, and 72 h for each treatment. All samples were immediately submerged in liquid nitrogen and saved at −80 °C until use. Each sample includes three biological replications.

### 2.9 RNA extraction and qRT‐PCR

Total RNA was extracted using the TRizol reagent (GenStar) and digested with DNaseI (TaKaRa) to remove DNA. The RNA was reverse transcribed to complementary DNA (cDNA) using RevertAid Reverse Transcriptase (Vazyme). Then the cDNA was diluted to 100 ng μl^−1^ with enzyme‐free water. The quantitative real‐time polymerase chain reaction (qRT‐PCR) analysis was carried out on a CFX 96 Real‐Time PCR system (Bio Rad) using SYBR Green Master Mix (Vazyme) following the manufacturer's instruction (Yin et al., 2021). Gene‐specific primers were designed using software Primer Premier 5 (Supplemental Table S1). The 20 μl. The qRT‐PCR reaction system consisted of 10 μl of 2 × SYBR Premix Extaq, forward and reverse primers 0.4 μl each, 2 μl diluted cDNA, and 7.2 μl ddH_2_O. The protocol was carried out as follows: predenaturation at 95 °C for 30 s (Step 1), denaturation at 95 °C for 5 s (Step 2), and primer annealing and extension and collection of fluorescence signal at 60 °C for 30 s (Step 3). The next 40 loops started in Step 2. Three technical replicates were used for each cDNA. Relative expression levels were determined using the 2^–ΔΔCt^ method (Yin et al., 2018). ADP‐ribosylation factor *Ta2291* (Forward: GCTCTCCAACAACATTGCCAAC, Reverse: GCTTCTGCCTGTCACATACGC), whose expression was stable under various conditions, was used as an internal reference gene for qRT‐PCR analysis (Huang et al., 2021).

### 2.10 Subcellular localization analysis and functional annotation of *TaLIM* genes

Full‐length primers for *TaLIM8‐4D* were designed using Primer Premier 5 (Forward: TTTGGAGAGGACACGCTCGAGATGTTCAGCGGGACGCAG, Reverse: ATAATCCATGAATTCCTCGAGGGAGGATTCAGCAGCCGC). Then, fragment was amplified by Phanta HS Master Mix (Vazyme) and inserted into the *Xho*I (NEB) digested vector pART27:GFP by using ClonExpress II One Step Cloning Kit (Vazyme). Fusion construct was transformed into *Agrobacterium tumefaciens* strain GV3101 and the pART27:GFP transformation was used as negative control. GV3101 carrying vectors were injected into *N. benthamiana* leaves (Zhu, Yang et al., 2019). Three days later, leaf fluorescence was visualized through confocal microscopy (TCS SP8) with 488 nm exciting light wavelength. Gene Ontology database was used to perform functional annotation of *TaLIM* genes (Zhu, Jiang et al., 2021).

### 2.11 Transient agroinfiltration assays

The pART‐27:TaLIM8‐4D:GFP fusion vectors were transient transformed into *N. benthamiana* plants by *A*. *tumefaciens* strain GV3101. Meanwhile, the pART27:GFP transformation was used as negative control. The agroinfiltration leaves were collected after 1, 2, 3, 4, and 5 d of treatment and quickly frozen in liquid nitrogen and stored at −80 °C for RNA extraction. *NbEF1a* was used as internal control gene for qRT‐PCR analysis. Then the relative expression levels of *PR* genes were calculated with the 2^–ΔΔCt^ method. Primer sequences were shown in Supplemental Table S2. Furthermore, the phenotype of pART‐27:TaLIM8‐4D:GFP in response to pathogen attack (*Phytophthora infestans* strain ‘88069’) was assessed. Briefly, *P*. *infestans* was cultured at 18 °C in darkness for 2 wk on rye sucrose agar (Yin et al., 2017). The sporangia suspension was prepared with 5 ml distilled water, and the concentration of the sporangia suspension was adjusted to 4 × 10^4^ sporangia ml^−1^ under the microscope. The spore suspension was placed in a refrigerator at 4 °C for 2 h to release zoospores. Agroinfiltration experiments were performed on leaves of 6‐ to 8‐wk‐old *N. benthamiana*. After 24 h, infiltrated *N. benthamiana* leaves were detached and transferred into moist sealed plastic trays and inoculated with 10 μl *P. infestans* zoospore suspension (100 zoospores μl^−1^) at the infiltration sites. The inoculated leaves were incubated at 18 °C in darkness. Photographs were taken under ultraviolet light and lesion diameters of the inoculated leaves were recorded 4 d after inoculated. Plants were cultivated in a greenhouse with temperature of 22–25 °C and under 16 vs. 8 h, day vs. night photoperiod (Gu et al., 2011). According to the former mentioned method, *TaLIM8‐4D* was transient overexpression in young wheat leaves by *A*. *tumefaciens* and GFP was used as negative control. After 3 d, qRT‐PCR was used to confirm the expression level of *TaLIM8‐4D*. Meanwhile, the suspension of *F. graminearum* spores were inoculated on the surface of overexpression and control leaves. The lesion sizes were recorded 4 d later. Assay consisted of three biological replications; each biological replication contained 10 leaves.

## 3 RESULTS

### 3.1 Identification and phylogenetic analysis of *LIM* genes in wheat genome

**FIGURE 1:**
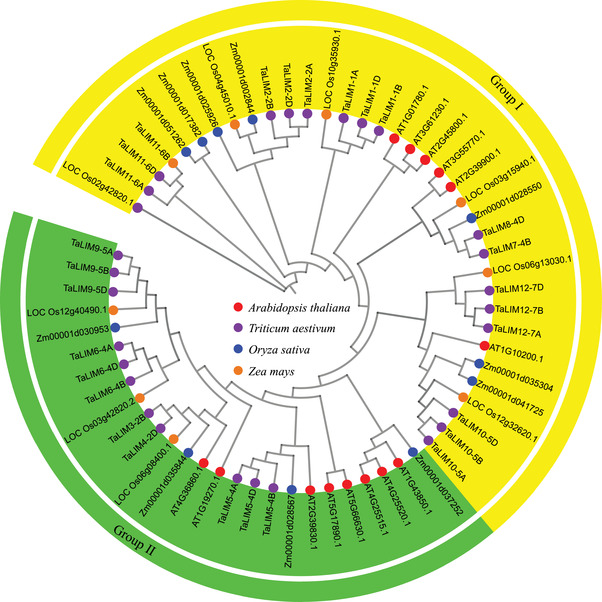
Phylogenetic tree of *Triticum aestivum*, *Zea mays*, *Oryza sati*va, and *Arabidopsis thaliana* Lin‐11, Isl‐1, and Mec‐3 domains (LIM) transcription factors

Twenty‐eight *TaLIM* genes were identified from wheat genome (Supplemental Table S1). To determine the evolutionary relationship between TaLIMs and AtLIMs, OsLIMs, and ZmLIMs, a phylogenetic tree containing 62 LIM protein sequences was constructed by multiple sequence alignment using ClustalW2. Based on the phylogenetic relationship, the 62 LIMs were divided into two groups: Group I with 36 LIMs and Group II with 26 LIMs (Figure 1). Among the 28 *TaLIM* genes, 17 have two LIM‐conserved domains and belong to Group I; 11 belong to Group II and contain one LIM domain at the N‐terminal and another new conserved cysteine‐rich domain DA1‐like at the C‐terminal. Those TaLIMs were named by comprehensive consideration of the evolutionary relationships, the character of conserved domains, and the chromosome position. The name of TaLIMs was shown in Supplemental Table S3.

### 3.2 Chromosomal mapping, gene duplication and Ka/Ks analysis of *TaLIM* genes

**FIGURE 2:**
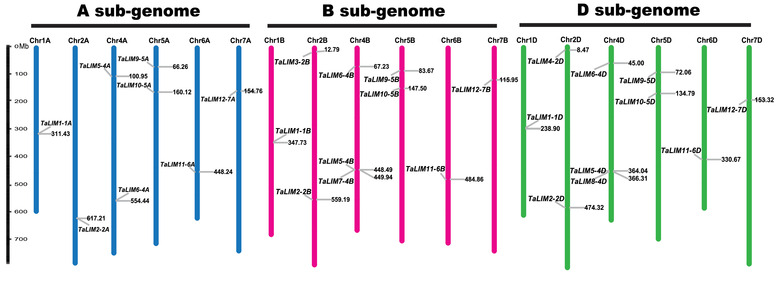
Chromosomal distribution of wheat (*Triticum aestivum*) Lin‐11, Isl‐1, and Mec‐3 domains (LIM) (*TaLIM*) genes. The ruler on the left represents the physical distance among genes (Mbp). The start and end information of 28 *TaLIM* genes on chromosomes was shown in Supplemental Table S5. Chr, chromosome

As shown in Figure 2, chromosome distribution map of *TaLIM* genes was constructed by using MapInspect according to the physical chromosome location extracted from the GFF3 file. The map showed that the 28 identified *TaLIM* genes were unevenly distributed on 18 chromosomes, whereas no *TaLIM* was located on chromosome 3. The number of *TaLIM* genes in A, B, and D subgenomes were counted. Results showed that there were eight *TaLIM* genes in A subgenome, 10 *TaLIM* genes in B subgenome, and 10 *TaLIM* genes in D subgenome. Based on the sequence similarity and chromosome position, a total of 26 pairs of fragment duplications were found among 28 *TaLIM* genes, and no tandem duplication event was found. The result suggested that fragment duplication is a major contributor to the expansion of LIM family in wheat during evolution. In order to further decipher the evolutionary history of *TaLIM* genes, we calculated the Ka/Ks values of 26 pairs of *TaLIM* fragment duplications using TBtools. Generally, the Ka/Ks value is widely used to represent the selection pressure and evolutionary speed of gene evolution. A Ka/Ks value of 1 represents the neutral selection effect, Ka/Ks value >1 represents positive selection of evolution acceleration, and Ka/Ks value <1 represents purifying selection under functional constraints. In this study, the Ka/Ks values of the 26 duplicate pairs were all <1 and the average value is 0.11 (Supplemental Table S4), indicating that they underwent strong purification selection in the evolutionary process, which may contribute to functional stability.

### 3.3 *TaLIM* genes were evolutionary conserved

**FIGURE 3:**
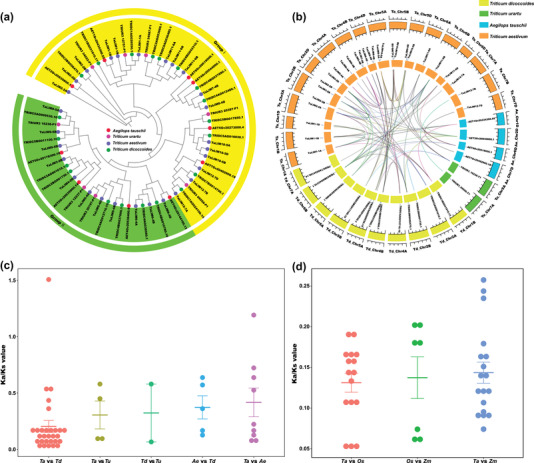
(a) Phylogenetic and (b) synteny analyses for *LIM* genes among *Triticum aestivum* and its ancestors *T*. *urartu*, *T*. *dicoccoides*, and *Aegilops tauschii*. Scatter diagram of nonsynonymous substitution rate (Ka)–synonymous substitution rate (Ks) ratio (Ka/Ks) values among (c) *T*. *aestivum* and three ancestors and (d) among *T*. *aestivum*, maize, and rice. The homologous pairs were showed in Supplemental Table S6

In order to further understand the evolutionary relationships of homology *LIM* genes between allohexaploid common wheat (AABBDD) and three ancestral species, BLASTp search was performed to identify homologous genes from red wild einkorn (AA), rough‐spike hard grass (DD), and wild emmer (AABB), which resulted in the detection of 8, 10, and 20 homologous *LIM* genes, respectively (Figure 3a). Furthermore, as showed in Supplemental Figure S1, almost all *LIM* genes were detected in the identical genomic regions of wheat A, B, and D subgenomes and the proposed ancestral A, B, and D subgenomes in wild einkorn, rough‐spike hard grass, and wild emmer, respectively. Therefore, it suggested that the *TaLIM* genes are possibly derived from ancestors and expanded by polyploidization after the crossing between ancestral species. To further confirm this speculation, the homologous gene pairs between wheat and ancestral species were identified, which resulted in the detection of 4, 11, and 36 homologous pairs between wheat and wild einkorn (Ta vs. Tu), wheat and rough‐spike hard grass (Ta vs. Ae), and wheat and wild emmer (Ta vs. Td), respectively (Figure 3b). Among the 51 homologous gene pairs, the Ka/Ks ratios for 49 pairs were <1 (Figure 3c), implying that most duplicated gene pairs underwent purification selection pressure, which helps their function to be maintained and reflects that they do not have much divergence during evolution. To further validate the conservation of LIMs, homologous genes between the Gramineae family species were identified and their Ka/Ks ratios were calculated. The result showed that 41 pairs of homologous genes among wheat, maize, and rice were identified. The Ka/Ks analysis showed that ratios for homologous pairs were <1 and the average value is 0.14 (Figure 3d; Supplemental Table S6), implying that the evolution of *LIM* genes were conserved in Gramineae family species. The above results indicate that *LIM* genes were not only conserved in the evolution of wheat but also highly conserved in grass plants.

### 3.4 Protein characterization and homology modeling of TaLIMs

In order to further determine the protein characteristic of TaLIMs, the online tool ExPASY Server10 was used to predict the protein properties of TaLIMs (Supplemental Table S1). Results showed that the amino acid length of TaLIMs ranged from 196 to 1,279 aa. The instability index showed that the majority of TaLIMs were unstable proteins (instability index > 40). Only Group I members TaLIM11‐6A, TaLIM11‐6B, TaLIM11‐6D, TaLIM1‐1A, TaLIM1‐1B, TaLIM1‐1D, TaLIM10‐5A, TaLIM10‐5B, and TaLIM10‐5D were predicted to be stable protein (instability index < 40). The TaLIMs isoelectric point varied significantly between 4.57 and 8.63, and there were both acidic and basic proteins. The grand average of hydropathicity of every TaLIM is <0, suggesting that they are all hydrophilic proteins. Since no signal peptides were detected, it was speculated that all TaLIMs were nonsecreted proteins. The subcellular localization prediction showed that all 11 TaLIMs in Group II were located in the nucleus. In Group I, 12 TaLIMs were located in the nucleus, while TaLIM11‐6A, TaLIM7‐4B, TaLIM8‐4D, TaLIM10‐5B, and TaLIM10‐5D were located in both nucleus and golgiosome.

**FIGURE 4:**
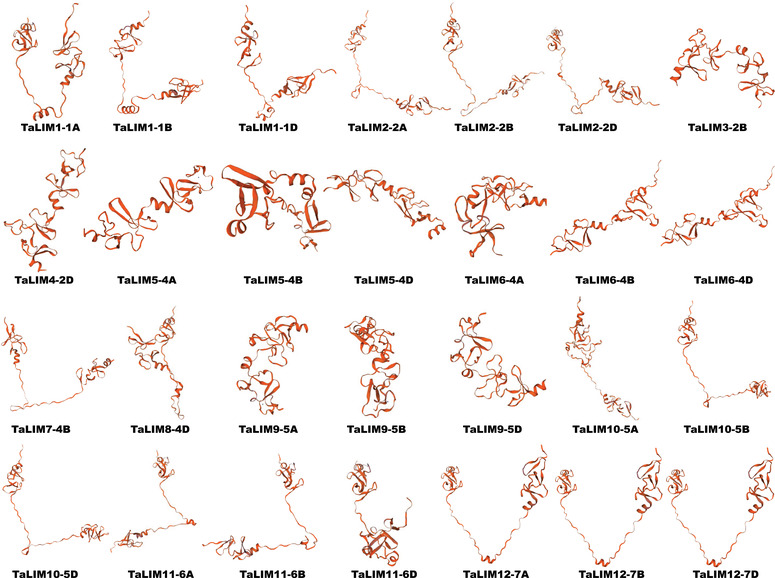
Predicted three‐dimensional models of wheat (*Triticum aestivum*) Lin‐11, Isl‐1, and Mec‐3 domains (LIM) (TaLIM) proteins. Models were generated by using online website SWISS‐MODEL (https://swissmodel.expasy.org)

The three‐dimension homology modeling of TaLIMs were completed by SWISS‐MODEL and results showed that TaLIMs are mainly comprised by *α*‐helix and *β*‐turn and have considerable simple three‐dimensional structures, whereas the proportion of these two kinds of structures are different among TaLIM proteins (Figure 4). Moreover, the proportion of *α*‐helix (20–38%) was greater than that of *β*‐turn (2.5–14%).

### 3.5 Gene structure and motifs analysis of *TaLIM* genes

**FIGURE 5:**
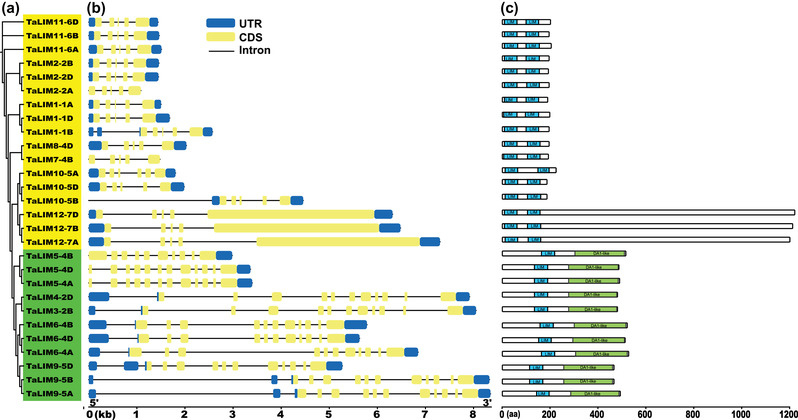
Exon–intron structure and conserved domain analyses. (a) Phylogenetic tree of 28 wheat (*Triticum aestivum*) Lin‐11, Isl‐1, and Mec‐3 domains (LIM) (*TaLIM*) genes. (b) Exon–intron analysis of 28 *TaLIM* genes. The blue and yellow box represent untranslated region (UTR) and coding sequence (CDS). The black line represents intron. (c) Diagram of LIM and DA1‐like conserved functional domains

**FIGURE 6:**
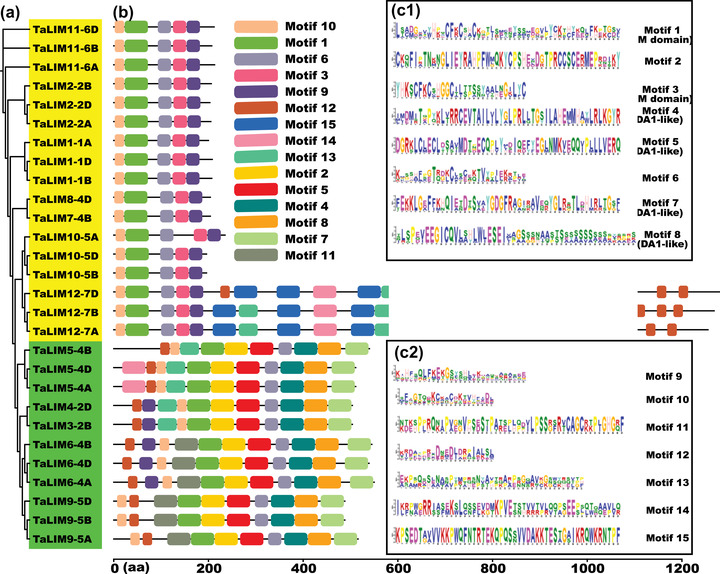
Conserved motifs of 28 wheat (*Triticum aestivum*) Lin‐11, Isl‐1, and Mec‐3 domains (LIM) (*TaLIM*) genes. (a) Phylogenetic tree of *TaLIM* genes. (b) Motif analysis of *TaLIMs*. Motifs were represented in different colors. (c) Conserved amino acid sequences of 15 motifs in *TaLIMs*

Gene structure analysis results showed that all *TaLIM* genes contained introns. The number of introns ranged from four to 12 and the number of exons ranged from five to 12. Most of the *TaLIM* genes (23) contained complete untranslated regions except for *TaLIM5‐4A*, *TaLIM5‐4B*, and *TaLIM5‐4D*, which only contained 3′‐UTR region, and *TaLIM2‐2D* and *TaLIM8‐4D* contained no UTR region (Figure 5b). To further study structural diversity and predict the function of TaLIM proteins, 15 motifs were identified using MEME online software and further annotated using Interproscan5 (Figure 6b). Detailed information of 15 motifs including specific amino acid sequences of each motif is shown in Supplemental Table S7. Meanwhile, WebLogo was used to generate a conservative design logo icon (Figure 6c). Results showed that TaLIM proteins closely clustered in neighboring phylogenetic branches (Figure 6a) have similar motif arrangements and positions, suggesting that members in same tree branch may have similar biological function. All the TaLIM proteins contained motif1 (LIM domain) and motif6. The function of motif6 is unknown. Group I members specifically contained motif3 (LIM domain), and LIM‐domain‐containing proteins have been shown to play roles in cytoskeletal organization and organ development (Kawaoka & Ebinuma, 2001; Maul et al., 2003). Group II members specifically contained motif4, 5, 7, 8, which comprise the DA1‐like conserved domain. But the function of DA1‐like domain is unknown.

### 3.6 Analysis of *cis*‐acting element for *TaLIM* genes

**FIGURE 7:**
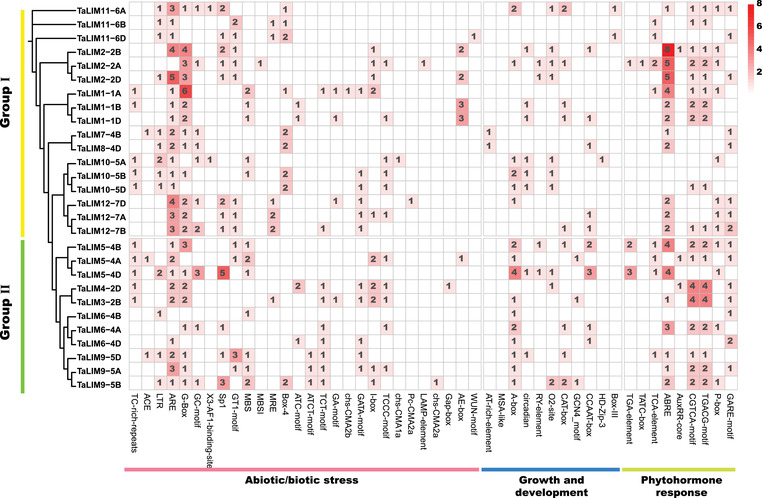
Statistical analysis of *cis*‐acting elements in the promoter of wheat (*Triticum aestivum*) Lin‐11, Isl‐1, and Mec‐3 domains (LIM) (*TaLIM*) genes. The color and number of the grid represent the number of *cis*‐acting elements in corresponding *TaLIMs*

Forty‐eight kinds of *cis*‐acting elements were detected in the upstream 1,500‐bp region of 28 *TaLIM* genes and these *cis*‐acting elements were divided into three groups: 28 *cis*‐acting elements belong to ‘biological–abiotic stress’, 11 belong to ‘growth and development’, and nine belong to ‘phytohormone response’ (Figure 7). The detailed information of these identified *cis*‐acting elements including function and location were displayed in Supplemental Table S8. Results showed that the promoter sequences of *TaLIM* genes have various *cis*‐regulatory elements, such as *cis*‐acting elements involved in defense and stress responsiveness, light‐responsive element, low‐temperature inducer, anaerobic‐induced *cis*‐regulating element, and drought‐inducing elements were found belonging to biological–abiotic group. The growth and development group contains cell‐cycle regulation, circadian‐rhythm control, and zein metabolism regulation related *cis*‐acting elements. And phytohormone response group contains gibberellins, jasmonic acid, ABA, and salicylic acid response related elements. The most *cis*‐acting elements are related to plant ABA (ABRE), which is distributed in the promoter region of most of *TaLIM* genes. The second most are anaerobic induction (ARE) elements, which are the most widely distributed and are present in almost all *TaLIM* genes. This suggests that the TaLIM proteins may be extensively involved in the anaerobic induced response and abscisic acid metabolism pathway.

### 3.7 Transcriptomic profiling revealed various expression patterns of *TaLIM* genes

**FIGURE 8:**
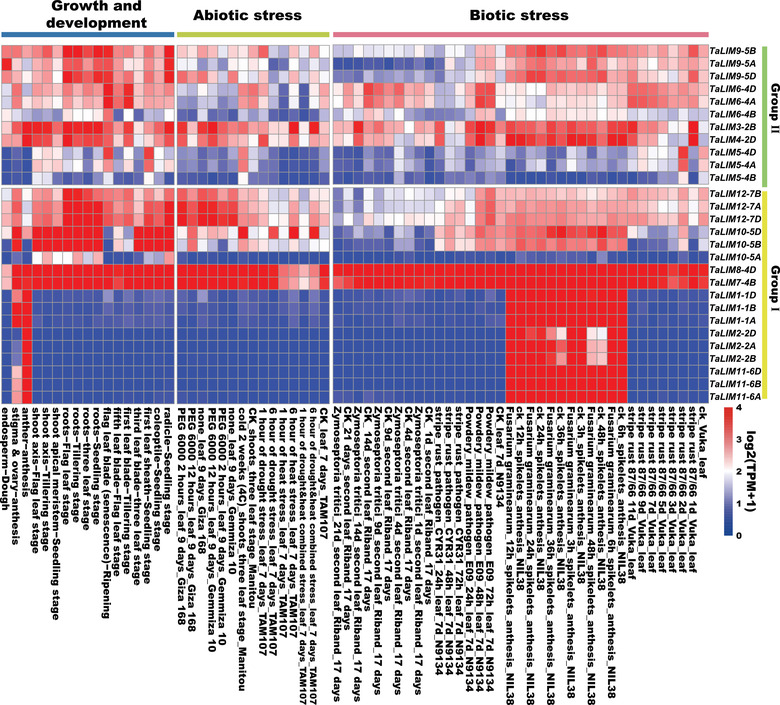
Expression pattern profiling of wheat (*Triticum aestivum*) Lin‐11, Isl‐1, and Mec‐3 domains (LIM) (*TaLIM*) genes under growth, development, and biotic and abiotic stresses determined by RNA sequencing analysis. The log_2_ (Transcripts Per Kilobase of exon model per Million mapped reads + 1) values were used to draw the heatmap

Expression patterns analysis showed that *TaLIM* genes have variable expression patterns. The expression levels of *TaLIM7‐4B* and *TaLIM8‐4D* were generally high throughout the growth period and under various biotic and abiotic stress conditions. Under various biotic stress, including *Zymoseptoria tritici*, powdery mildew, stripe rust, and *F*. *graminearum*, the expression levels of 17 *TaLIM* genes were highly induced. Whereas the expression levels of *TaLIM1‐1A*, *TaLIM1‐1B*, *TaLIM1‐1D*, *TaLIM2‐2A*, *TaLIM2‐2B*, *TaLIM2‐2D*, *TaLIM11‐6A*, *TaLIM11‐6B*, and *TaLIM11‐6D* were only highly induced under the *F. graminearum* treatment. As shown in Figure 8, under 17 different growth and development stages, the expression levels of 19 *TaLIM* genes were highly induced, whereas *TaLIM1‐1A*, *TaLIM1‐1B*, *TaLIM1‐1D*, *TaLIM11‐6A*, *TaLIM11‐6B*, and *TaLIM11‐6D* were highly expressed only in anther–anthesis stage and stigma and ovary‐anthesis stage. Genes *TaLIM2‐2A*, *TaLIM2‐2B*, and *TaLIM2‐2D* were highly expressed only in the anther–anthesis stage. Under abiotic stress, most of the genes were expressed, whereas the expression levels were low (Supplemental Table S9). Genes *TaLIM2‐2A* and *TaLIM11‐6D* were not expressed, while *TaLIM2‐2B*, *TaLIM2‐2D*, *TaLIM11‐6A*, and *TaLIM11‐6B* were expressed only under certain treatments such as *TaLIM2‐2B* and *TaLIM11‐6A* under *F. graminearum* treatment. Moreover, 28 *TaLIM* genes were highly expressed at anther–anthesis stage, suggesting potential involving roles of *TaLIM* genes in pollen tube growth of wheat, which also conforms to the function of *LIM* genes.

### 3.8 Expression analyses of *TaLIM* genes

**FIGURE 9:**
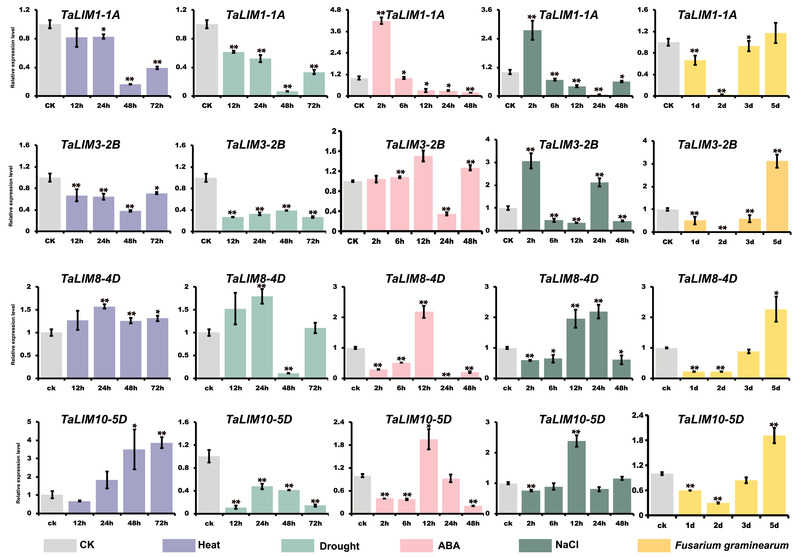
Expression patterns of four wheat (*Triticum aestivum*) Lin‐11, Isl‐1, and Mec‐3 domains (LIM) (*TaLIM*) genes under various treatments. The treatments included heat, drought, 150 mM NaCl, 100 μM abscisic acid, and *Fusarium graminearum*. The *y* axis represents the relative expression levels, and the *x* axis shows the time points of stresses treatment. *Significant at the .05 probability level. **Significant at the .01 probability level

To further understand the role of *TaLIM* genes in response to biological and abiotic stresses, the transcriptomic profiles were considered (Figure 8) and several highly expressed (*TaLIM8‐4D*) and differentially expressed (*TaLIM1‐1A*, *TaLIM3‐2B*, and *TaLIM10‐5D*) *LIM* genes were selected to analysis their expression patterns in responding to *F. graminearum*, heat, drought, ABA, and NaCl stresses using qRT‐PCR (Figure 9).

In the pollen, under heat treatment, the expression levels of *TaLIM1‐1A and TaLIM3‐2B* were downregulated, with the expression levels being the lowest after 48 h. The expression levels of *TaLIM8‐4D* and *TaLIM10‐5D* were upregulated after 12, 24, 48, and 72 h of treatment. The expression levels of *TaLIM10‐5D* were upregulated after 24, 48, and 72 h of treatment, but the expression level was decreased after 12 h of treatment. Under drought treatment, the expression levels of *TaLIM1‐1A*, *TaLIM3‐2B*, *and TaLIM10‐5D* in the pollen were downregulated. The expression levels of *TaLIM8‐4D* were upregulated after 12, 24, and 72 h of treatment, but after 48 h of treatment, the expression level was downregulated compared with control.

In the leaves, under ABA treatment, the expression levels of *TaLIM1‐1A* were upregulated after 2 h of treatment but downregulated after 6, 12, 24, and 48 h of treatment. The expression levels of *TaLIM8‐4D* and *TaLIM10‐5D* were downregulated after 2, 6, 24, and 48 h of treatment but upregulated after 12 h of treatment. The expression levels of *TaLIM3‐2B* were upregulated after 2, 6, 12, and 48 h of treatment but downregulated after 24 h of treatment.

In the leaves, under NaCl treatment, the expression levels of *TaLIM1‐1A* were upregulated after 2 h of treatment but downregulated after 6, 12, 24, and 48 h of treatment. The expression levels of *TaLIM3‐2B* were upregulated after 2 and 24 h of treatment but downregulated after 6, 12, and 48 h of treatment. The expression levels of *TaLIM8‐4D* were downregulated after 2, 6, and 48 h of NaCl treatment but upregulated after 12 and 24 h of treatment. The expression levels of *TaLIM10‐5D* were downregulated after 2, 6, and 24 h of treatment but upregulated after 12 and 48 h of treatment.

In the coleoptiles, under the treatment of *F. graminearum*, the expression levels of *TaLIM1‐1A*, *TaLIM3‐2B*, *TaLIM8‐4D*, and *TaLIM10‐5D* were downregulated after 1, 2. and 3 d of treatment but upregulated after 5 d of treatment. Furthermore, the expression levels of *TaLIM1‐1A* and *TaLIM3‐2B* were lowest after 2 d of treatment.

### 3.9 GO annotation and subcellular localization of TaLIM proteins

**FIGURE 10:**
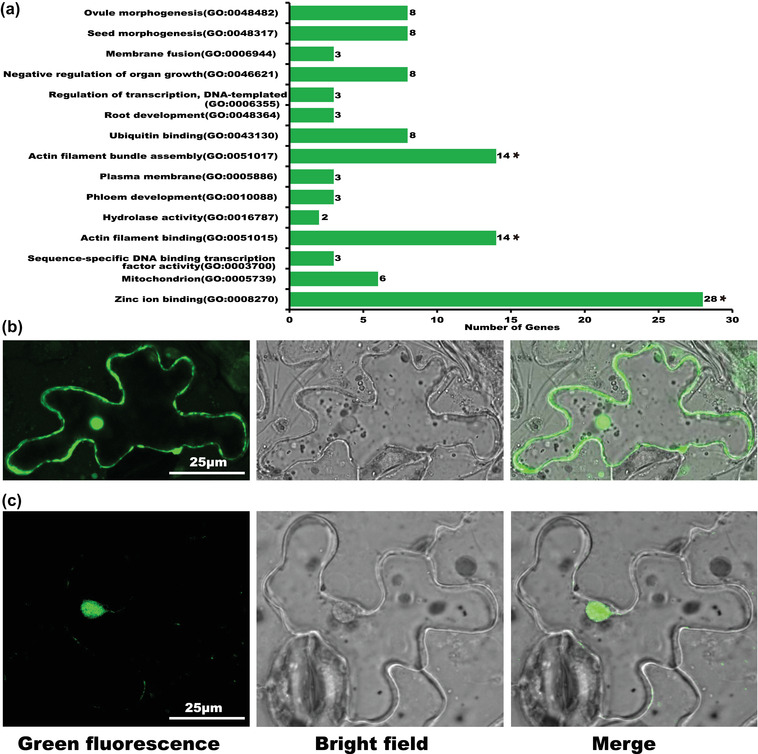
Gene Ontology (GO) annotation and the subcellular localization of *TaLIM8‐4D*. (a) Gene Ontology annotation of *TaLIM* genes. An asterisk (*) indicates *TaLIM8‐4D* was annotated under the GO terms. (b) Subcellular localization of free green fluorescent protein. (c) Subcellular localization of *TaLIM8‐4D*. Photographs were taken using confocal microscopy. Scale bar is 25 μm

TaLIM protein functions were predicted by GO annotation. As shown in Figure 10a (detailed information listed in Supplemental Table S10), all TaLIMs (28) were annotated under GO terms ‘zinc ion binding’ (GO:0008270). Half of TaLIMs (14) were annotated under ‘actin filament binding’ (GO:0051015) and ‘actin filament bundle assembly’ (GO:0051017). The results implying TaLIM proteins could be an actin binding protein that are involved in various developmental activities and cellular processes in plants as suggested by previous studies (Thaler et al., 2002). Moreover, some triplets of TaLIM proteins have the same function annotation. such as TaLIM10‐5A, TaLIM10‐5B. and TaLIM10‐5D, were annotated under ‘sequence‐specific DNA binding transcription factor activity’, ‘plasma membrane’, ‘regulation of transcription, DNA‐templated’ and ‘membrane fusion’. TaLIM5‐4A, TaLIM5‐4B and TaLIM5‐4D were annotated under ‘phloem development’ and ‘root development’. Consistently, with the function annotation, the subcellular localization prediction showed that 28 TaLIM proteins were localization in the nucleus, while TaLIM11‐6A, TaLIM7‐4B, TaLIM8‐4D, TaLIM10‐5B, and TaLIM10‐5D were predicted to be located in the nucleus and golgiosome. To verify subcellular localization prediction and reveal the possible functions of TaLIM proteins, we constructed the GFP fusion protein expression vector pART‐27:TaLIM8‐4D:GFP to do the subcellular analysis. Compared with the control (Figure 10b), TaLIM8‐4D is localization in the nucleus (Figure 10c). These results suggest that TaLIM8‐4D plays a biological role in the nucleus.

### 3.10 TaLIM8‐4D promoted the infection of pathogens

**FIGURE 11:**
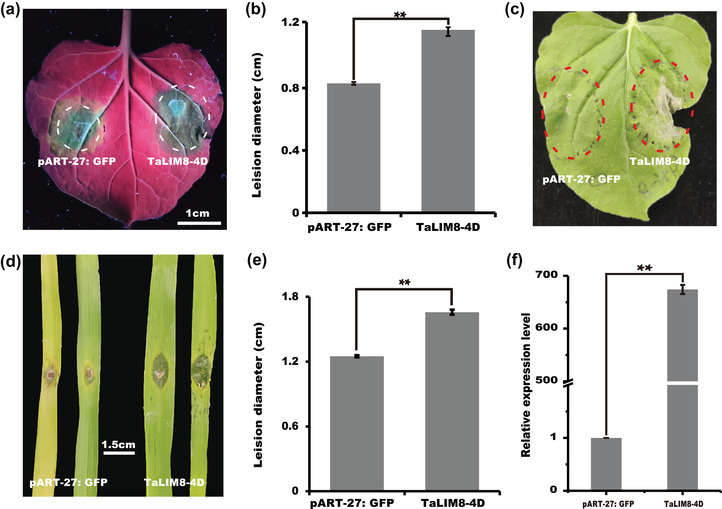
Function analysis of *TaLIM8‐4D*. (a) Overexpression of *TaLIM8‐4D* promoted *Phytophthora infestans* colonization. Photographs were taken under ultraviolet headlights. The white circle represents the area of the lesion. Scale bar is 1 cm. (b) Lesion diameter of control (green fluorescent protein [GFP]) and *TaLIM8‐4D*. (c) *TaLIM8‐4D* induced weak cell death. (d) Overexpression of *TaLIM8‐4D*‐promoted *Fusarium graminearum* colonization. Scale bar is 1.5 cm. (e) Lesion diameter of control (GFP) and *TaLIM8‐4D*. (f) Relative expression level of *TaLIM8‐4D* in transient overexpression plants. **Significant at the .01 probability level

To preliminarily test the biological function and reveal whether *TaLIM8‐4D* participates in the pathogenesis process during pathogen infection host, this gene was cloned and transiently expressed in *N. benthamiana* using agrobacterium‐mediated expression followed by a hemibiotrophic pathogen *P. infestans* infection. As shown in Figure 11a and 11b, the result suggested *TaLIM8‐4D* promoted the colonization of *P. infestans*, implying *TaLIM8‐4D* function as a susceptible gene. Meanwhile, weak cell death was observed in *N*. *benthamiana* leaf after 5 d transient expression, indicating that *TaLIM8‐4D* is a cell‐death inducer (leave cell death as shown in Figure 11c). After overexpression of *TaLIM8‐4D* in young wheat leaves for 3 d, qRT‐PCR analysis was used to confirm the expression level of *TaLIM8‐4D*, and results showed that the *TaLIM8‐4D* was overexpressed for 674‐fold when compared with control (Figure 11f). Then suspension of *F. graminearum* spores were inoculated on the surface of leaves. After 4 d, the lesion sizes were recorded and results showed that, *TaLIM8‐4D* overexpression significantly promoted the infection of *F*. *graminearum*, as the lesion size was 33% larger than control (Figure 11d and 11e). These results suggested that *TaLIM8‐4D* was a susceptible gene and its overexpression can promote the infection of *F*. *graminearum*.

### 3.11 *PR* genes were upregulated by *TaLIM8‐4D*

**FIGURE 12:**
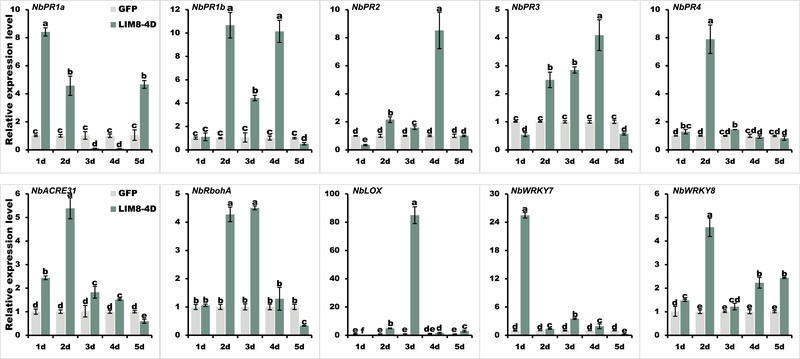
Relative expression level of *pathogenesis*‐*related* (*PR*) genes in transient overexpression of *TaLIM8‐4D* plants. Each bar represents the average of three replicates, and the error bar represents standard deviation (SD). Different letters in a column indicate significant differences between the treatments at *p* < .05 level

To explore the influence of TaLIM8‐4D on the expression of *PR* genes, *TaLIM8‐4D* was transiently overexpressed in *N. benthamiana* leaves. The expression of three Pattern Triggered Immunity‐related genes (*NbWRKY7*, *NbWRKY8*, and *NbACRE31*), one reactive oxygen‐related gene (*NbRbohA*), three salicylic acid‐response genes (*NbPR1a*, *NbPR1b*, and *NbPR2*), and three jasmonic acid‐dependent immunity genes (*NbPR3*, *NbPR4*, and *NbLOX*) were analyzed. As shown in Figure 12, *PR* genes, such as *NbPR1b*, *NbACRE31*, *NbWRKY7*, and *NbRbohA*, were upregulated at 1–4 d in *TaLIM8‐4D* overexpression plant but downregulated at 5 d. The expression of *NbPR3*, *NbPR4*, *NbPR2*, and *NbLOX* were upregulated at a specific time point. For example, *NbLOX* and *NbPR2* were downregulated at 1 d but upregulated at 2–5 d. *NbPR4* was upregulated at 1–3 d but downregulated at 4–5 d; *NbPR3* was downregulated at 1 and 5 d but upregulated at 2–4 d. These results demonstrate the *TaLIM8‐4D* genes could trigger the expression of *PR* genes to activate the hypersensitive response (Figure 11c), which might be the reason why the larger disease lesion was observed in *TaLIM8‐4D* transgenic plant as shown in Figure 11a,b,d,e. Integrating considering the results of *TaLIM8‐4D* overexpression and pathogen inoculation assays, it was speculated that *TaLIM8‐4D* was function as susceptible gene in nucleus by upregulating *PR* genes and inducing weak cell death to promote the colonization of hemibiotrophic agent *F. graminearum*. However, this function mechanism needs to be further confirmed using wheat stale transformants.

## 4 DISCUSSION

The LIM proteins, which act as developmental regulators in basic cellular processes such as regulating the transcription or organizing the cytoskeleton, are widely found in eukaryotes (Arnaud et al., 2007). Considering their important roles, the genome‐wide identification of LIMs has been conducted in several plant species such as rice, black cottonwood, *Arabidopsis* (Arnaud et al., 2007), maize (Zhang et al., 2013), and quinoa (Zhu, Wang et al., 2021). Whereas there still lacks a systemic survey of LIM family in wheat. In order to get insights into the function of wheat LIMs, comprehensive analysis of TaLIMs was performed in the present study.

For family member identification, known LIM protein sequences and conserved domain sequences were generally used as seed sequences to perform BLASTp searching (He et al., 2020). Accordingly, we collected 14 AtLIMs, nine OsLIMs, and 11 ZmLIMs from *Arabidopsis* (Arnaud et al., 2007), rice (Arnaud et al., 2007) and maize (Zhang et al., 2013) and downloaded LIM domain sequences (PF00412) from Pfam. Both sequences were queried against wheat genome (IWGSC v2.1) (reference) and candidate hits were further checked by Pfam and InterProScan to exclude sequences without LIM domain. Finally, 28 wheat LIM proteins were identified and named including 17 double LIM domain proteins and 11 single LIM domain proteins. Single LIM domain protein only retains the LIM domain at the N‐terminal, and a new highly conserved domain DA1‐like at the C‐terminal (Figure 5c). Although the function is unknown, DA1‐like domain is rich in cysteine residues, which is similar to LIM domain and believed it may be the mutation of LIM (Zhu, Wang et al., 2021).

Gene copy number variation is the key foundation for the plant genome adaptation to the variable environment since each copy of the gene can evolve independently and diversify its functions (Kondrashov, 2012). Fragment duplication and tandem duplication are generally considered to be the two main causes of gene copy number variation, which further result in the extension of plant gene family (Zhu et al., 2014). Therefore, it is necessary to detect the duplication events of *TaLIM* genes. In this study, based on the sequence similarity and chromosome position, 26 pairs of fragment duplications were found but no tandem duplication was detected, implying that fragment duplication is the dominant reason for the expansion of *TaLIM* family during evolution. To further reveal the selection pressure, Ka/Ks ratios were calculated for 26 pairs of duplicated genes. As showed in Supplemental Table S4, all ratios were less than one and the average values was 0.11, suggesting that *TaLIM* possibly underwent strong purification selection in the evolutionary process, which may contribute to functional stability. To further validate the purification selection, we expanded the duplication analysis and Ka/Ks ratio calculation to wheat and ancestor spices (Supplemental Table S6). Previous research showed that the evolutionary history of common wheat is quite complex, and its ancestral origin is influenced by many factors. But it is commonly believed that red wild einkorn (wild diploid wheat) crossed with the B genome ancestor that is the closest relative of *Ae. speltoides* Tausch to produce wild emmer wheat (Dvorak & Akhunov, 2005; Huang et al., 2002; Peng et al., 2011). To further understand the evolutionary relationship and homology of the *LIM* genes among allohexaploid common wheat (AABBDD) and ancestral species, we used BLASTp to identify homologous genes from red wild einkorn (AA), rough‐spike hard grass (DD), and wild emmer (AABB), which resulted in the detection of 8, 10, and 20 homologous genes, respectively (Figure 3a). Furthermore, as shown in Supplemental Figure S1, almost all *LIM* genes were detected in the identical genomic regions of wheat A, B, and D subgenomes and the corresponding A, B, and D subgenomes belonging to the contributing ancestors. Therefore, we speculate that the *TaLIM* genes possibly derived from ancestors and expanded by polyploidization after the crossing between ancestral species. To further confirm this speculation, we identified the homologous gene pairs between common wheat and ancestral species, resulting in the detection of 4, 11, and 36 homologous pairs between wheat and red wild einkorn (Ta vs. Tu), wheat and rough‐spike hard gras (Ta vs. Ae), and wheat and wild emmer (Ta vs. Td), respectively (Figure 3b). Among the 51 homologous gene pairs, the Ka/Ks ratios for 49 pairs were <1 (Figure 3c), implying that most duplicated gene pairs underwent purification selection pressure, which helps their function to be maintained and reflects that they do not have much divergence during evolution. To further validate the conservation of *LIM* genes, we identified homologous genes between the Gramineae family species and calculate their Ka/Ks values. The result showed that 41 pairs of homologous genes among wheat, maize, and rice were identified. The Ka/Ks analysis showed that ratios for homologous pairs were <1 and the average value is 0.14 (Figure 3d; Supplemental Table S6), implying that the evolution of *LIM* genes were conserved in Gramineae family species. The above results indicate that *LIM* genes were not only conserved in the evolution of wheat, but also highly conserved in grass plants, thus further indicating that genome polyploidization promoted the expansion of TaLIM family.

Structure determines function and exon–intron structural diversity usually play key roles in the evolution of gene families, which can provide additional evidence to support phylogenetic grouping (Shiu & Bleecker, 2003). Therefore, we used the GFF3 file of *TaLIM* genes to analyze their exon–intron structure and was illustrated by GSDS visualization server. Results showed that all *TaLIM* genes contained introns, but the number of introns is different, which may be one of the reasons for the functional differentiation of *TaLIM* genes. Protein conserved functional domain analysis showed that all of the 17 members in Group I contain two LIM domains, and all of the 11 members in Group II contain one LIM domain and one DA1‐like domain, which is consistent with previous studies (Figure 5a,c) (Arnaud et al., 2012). Protein motif prediction is an effective method to predict protein function (Jiang et al., 2020). In order to further study structural diversity and predict the function of TaLIM proteins, 15 motifs were identified using MEME online software and further annotated using Interproscan5 (Figure 6b). Results showed that TaLIM proteins closely clustered in phylogenetic tree (Figure 6a) have similar motif arrangements and positions, suggesting that the motifs between Group I and II members were different, while the members belong to same group contain similar motifs, which imply that the functions of different group members might be related to specific motif (Mukesh et al., 2007).

Transcription factors are important in the regulation of plant growth and development, hormone metabolism, and stress responses. While *cis*‐elements existed in the promoter region play important roles in the transcriptional level of genes in response to multiple stimuli (Yin et al., 2020). Therefore, the analysis of promoter *cis*‐acting elements is important in the study of genes function (Wang et al., 2020). In this study, through analysis, we observed that TaLIM genes contain many *cis*‐acting elements related to abiotic and biotic stresses such as *cis*‐acting element involved in light responsiveness (ACE) and *cis*‐acting regulatory element essential for the anaerobic induction (ARE). Meanwhile, we have also observed elements related to hormones (ABA, methyl jasmonate, gibberellin, salicylic acid, and auxin). These elements play a key role in the plant response to biotic and abiotic stresses and plant signal transduction (Wang et al., 2011). For example, ABA plays a vital role in plant growth and development as well as in mediating plant response to a wide range of stresses. The ABA response element (ABRE) was identified in most of *TaLIM* genes (17 genes), which interact with b‐ZIP transcription factors AREB1, AREB2, and ABF3 to jointly regulate plant response to osmotic stress (June‐Sik et al., 2011). Moreover, the other *cis*‐elements involved in osmotic stress, such as MBS and TC‐rich repeats, were also observed in *TaLIM* gene promoters. This result suggested that *LIM* genes in wheat may be regulated by various factors including drought and ABA, which need to be experimentally explored in further studies.

For multigene family, analyses of gene expression patterns often provided useful clues for determining gene function (Jiang et al., 2019). Data from a comprehensive study of wheat expression profiles containing 69 different situations were analyzed to reveal the expression patterns of 28 *TaLIM* genes (Supplemental Table S9) (Trapnell et al., 2012). The results revealed variable regulation models of *TaLIM* genes in different plant development stages and environment situations (Figure 8). The transcriptome data sets downloaded from NCBI were divided into three categories according to biotic stress, abiotic stress, and growth and development. Transcriptomic profiling analysis revealed that *TaLIM* genes showed variable expression patterns. Plants have evolved kinds of ways to protect themselves from abiotic and biotic stresses, which is mainly reflected in the expression of stress response genes (Zhou et al., 2018), therefore the stimulation‐specific expression of gene is closely related to its function. Meanwhile, previous studies have shown that *LIM* family genes play an important role in lignin synthesis, pollen tube growth, mechanical damage repair, organ development, and plant morphogenesis (Mundel et al., 2000). In this study, 28 *TaLIM* genes were expressed in various growth and development stages, and their expression levels were different in different stage (Figure 8; Supplemental Table S9), suggesting that *TaLIM* genes might be involved in the regulation of different growth and development stages of wheat. Moreover, 28 *TaLIM* genes were highly expressed at anther–anthesis stage, suggesting that *TaLIM* genes may be related to pollen tube growth of wheat, which also conforms to the function of *LIM* genes (Arnaud et al., 2012).

In order to understand the expression of *TaLIM* genes in response to stresses, we selected four highly expressed genes for qRT‐PCR analysis. The results show that *TaLIM* genes can participate in the response process of wheat to stresses including heat, drought, NaCl, ABA, and *F. graminearum* conditions (Figure 9). Previous study had shown that the *LIM* genes of tomato can be induced by ABA, cold, drought, NaCl, and heat treatments (Khadiza et al., 2016). Similar results were also found in this study. Furthermore *TaLIM8‐4D* was upregulated under treatments of *F. graminearum*, heat, drought, ABA, and NaCl, suggesting that *TaLIM8‐4D* could play an important role in regulating plant growth under abiotic and biotic stress (Figure 9). Following, in this study, the biological function of TaLIM8‐4D was systemically analyzed. RNA sequencing profiling and qRT‐PCR analysis both showed that *TaLIM8‐4D* was upregulated by the infection of hemibiotrophic agent *F. graminearum*, implying it was induced by the pathogen. Besides, consistent with the prediction, TaLIM8‐4D was experimentally observed in the nucleus (Figure 10), suggesting its transcription factor function. Additionally, TaLIM8‐4D is a cell‐death inducer because the weak cell death was observed after overexpression in *N. benthamiana* leaves for 5 d (Figure 11) (Yin et al., 2020). Furthermore, overexpression of *TaLIM8‐4D* can upregulate plant *PR* genes, such as Pattern Triggered Immunity‐related gene *NbWRKY8*, reactive oxygen‐related gene *NbRbohA*, salicylic acid‐response gene *NbPR1b*, and jasmonic acid‐dependent immunity gene *NbPR3*, and promote the infection of hemibiotrophic pathogen (Figure 12). Considering these results, it was speculated that *TaLIM8‐4D* was functioning as a susceptible gene in the nucleus by upregulating *PR* genes and inducing cell death to promote the colonization of hemibiotrophic agent *F. graminearum*. However, this function mechanism needs to be experimentally confirmed and, currently, we are constructing the stable transformant plants of *TaLIM8‐4D* to further validate its function.

## 5 CONCLUSIONS

In conclusion, 28 *TaLIM* genes were identified in wheat and were grouped into two groups. Group I members contain two LIM domains and Group II members contains one LIM and one DA1‐like domains. They are unevenly distributed on 18 chromosomes. Wheat LIMs are hydrophilic proteins and most of them are unstable. They are mainly comprised by α‐helix and β‐turn and without signal peptides. Polyploidization played a fundamental role in the expansion of TaLIM members because (a) TaLIM proteins showed fragment duplication but no tandem duplication, (b) they were under strong purification selection and evolutionary conserved among ancestral species even among Gramineae family, and (c) identical LIM orthologues were found in similar genomic regions of wheat A, B, and D subgenomes and their corresponding ancestral A, B, and D subgenomes. Wheat LIMs are functionally annotated to GO term ‘zinc ion binding’ and predicted to be localized in the nucleus. Wheat *LIM* genes showed variable expression patterns and certain members are responsive to biotic and abiotic stresses and growth and development conditions. Genes *TaLIM1‐1A*, *TaLIM3‐2B*, *TaLIM8‐4D*, and *TaLIM10‐5D* were significantly induced by heat, drought, NaCl, ABA, and *Fusarium graminearum* stresses. Furthermore, the biological function of TaLIM8‐4D was systemically analyzed and results revealed that TaLIM8‐4D showed nucleus localization and is induced by hemibiotrophic agent *F*. *graminearum*. It is a weak cell‐ death inducer and can upregulate the expression of *PR* genes and promote the infection of hemibiotrophic pathogen. Summarily, *TaLIM8‐4D* could function as susceptible gene in the nucleus through upregulating *PR* genes and inducing cell death to promote the colonization of hemibiotrophic *F*. *graminearum*.

## AUTHOR CONTRIBUTIONS

Yiting Li: Conceptualization; Methodology; Software; Writing – original draft; Writing – review & editing. Xi Liu: Methodology; Software. Yongxin Xiao: Methodology; Software. Yong Wen: Methodology. Keke Li: Software. Zhaolan Ma: Methodology. Lijun Yang: Methodology. Yongxing Zhu: Writing – review & editing. Junliang Yin: Funding acquisition; Project administration; Supervision; Validation; Writing – review & editing.

## CONFLICT OF INTEREST

The authors declare that they have no conflict of interest.

## Supporting information

Supplemental Figure S1. Chromosomal distribution of LIMs of *Triticum aestivum* (Ta), *Triticum urartu* (Tu), *Triticum dicoccoides* (Td), and *Aegilops tauschii* (Ae).

Supplemental Table S1. qRT‐PCR primers for *TaLIM* genes.
Supplemental Table S2. The primer sequences of pathogen response genes for qRT‐PCR.
Supplemental Table S3. Protein characterization for TaLIMs.
Supplemental Table S4. Ka/Ks analysis and estimated divergence time for duplicated TaLIMs.
Supplemental Table S5. Chromosome locations of *TaLIM* genes in the Chinese Spring wheat reference genome.
Supplemental Table S6. The homologous pairs of LIMs.
Supplemental Table S7. Multilevel consensus sequences in *TaLIM* genes identified by MEME.
Supplemental Table S8. The *cis*‐elements in upstream sequences of *TaLIM* genes.
Supplemental Table S9. Expression level of wheat *LIM* genes under multiple conditions.
Supplemental Table S10. Functional annotation of wheat *LIM* genes.

## Data Availability

All datasets supporting the conclusions of this article are included within the article (and its Supplemental material). The genome data and sequences and expression profiles of *TaLIMs* genes used in the current study are available in the Wheat Whole Genome Database (https://wheat‐urgi.versailles.inra.fr/Seq‐Repository/Assemblies). The datasets generated and analyzed during the current study are available from the corresponding author on reasonable request.

## References

[tpg220246-bib-0001] Arnaud, D. , Déjardin, A. , Leplé, J. C. , Lesage‐Descauses, M. C. , Boizot, N. , Villar, M. , Bénédetti, H. , & Pilate, G. (2012). Expression analysis of LIM gene family in poplar, toward an updated phylogenetic classification. BMC Research Notes, 5, 102. 10.1186/1756-0500-5-102 22339987 PMC3392731

[tpg220246-bib-0002] Arnaud, D. , Déjardin, A. , Leplé, J. C. , Lesage‐Descauses, M. C. , & Pilate, G. (2007). Genome‐wide analysis of LIM gene family in *Populus trichocarpa*, *Arabidopsis thaliana*, and *Oryza sativa* . DNA Research, 14, 103–116. 10.1093/dnares/dsm013 17573466 PMC2779900

[tpg220246-bib-0003] Chen, C. , Chen, H. , Zhang, Y. , Thomas, H. R. , & Xia, R. (2020). TBtools: An integrative toolkit developed for interactive analyses of big biological data. Molecular Plant, 13, 1194–1202. 10.1016/j.molp.2020.06.009 32585190

[tpg220246-bib-0004] Chen, F. , Hu, Y. , Vannozzi, A. , Wu, K. C. , Cai, H. Y. , Qin, Y. , Mullis, A. , Lin, Z. G. , & Zhang, L. S. (2017). The WRKY transcription factor family in model plants and crops. Critical Reviews in Plant Sciences, 36, 311–335. 10.1080/07352689.2018.1441103

[tpg220246-bib-0005] Dawid, I. B. , Breen, J. J. , & Toyama, R. (1998). LIM domains: Multiple roles as adapters and functional modifiers in protein interactions. Trends in Genetics, 14, 156–162. 10.1016/S0168-9525(98)01424-3 9594664

[tpg220246-bib-0006] Dawid, I. B. , Toyama, R. , & Taira, M. (1995). LIM domain proteins. Encyclopedia of Inorganic and Bioinorganic Chemistry, 318, 295–306. 10.1002/9781119951438.eibc0489 7788499

[tpg220246-bib-0007] Dvorak, J. , & Akhunov, E. (2005). Tempos of gene locus deletions and duplications and their relationship to recombination rate during diploid and polyploid evolution in the *Aegilops‐Triticum* alliance. Genetics, 171, 323–332. 10.1534/genetics.105.041632 15996988 PMC1456522

[tpg220246-bib-0008] Eliasson, A. , Gass, N. , Mundel, C. , Baltz, R. , Krauter, R. , Evrard, J. L. , & Steinmetz, A. (2000). Molecular and expression analysis of a LIM protein gene family from flowering plants. Molecular Genetics and Genomics, 264, 257–267. 10.1007/s004380000312 11085265

[tpg220246-bib-0009] Fang, Z. W. , He, Y. Q. , Liu, Y. K. , Jiang, W. Q. , Song, J. H. , Wang, S. P. , Ma, D. F. , & Yin, J. L. (2020). Bioinformatic identification and analyses of the non‐specific lipid transfer proteins in wheat. Journal of Integrative Agriculture, 19, 1170–1185. 10.1016/S2095-3119(19)62776-0

[tpg220246-bib-0010] Fang, Z. W. , Jiang, W. Q. , He, Y. Q. , Ma, D. F. , Liu, Y. K. , Wang, S. P. , Zhang, Y. X. , & Yin, J. L. (2020). Genome‐wide identification, structure characterization, and expression profiling of Dof transcription factor gene family in wheat (*Triticum aestivum* L.). Agronomy, 10, 294. 10.3390/agronomy10020294

[tpg220246-bib-0011] Gao, L. (2016). Effects of PEG‐6000 on physiological and biochemical characteristics of celery seedlings. Journal of Anhui Agricultural Sciences, 44, 12–14. 10.13989/j.cnki.0517-6611.2016.33.004

[tpg220246-bib-0012] Gu, B. , Kale, S. D. , Wang, Q. , Wang, D. , Pan, Q. , Cao, H. , Meng, Y. , Kang, Z. , Tyler, B. M. , & Shan, W. (2011). Rust secreted protein Ps87 is conserved in diverse fungal pathogens and contains a RXLR‐like motif sufficient for translocation into plant cells. PLoS One, 6, e27217. 10.1371/journal.pone.0027217 22076138 PMC3208592

[tpg220246-bib-0013] He, Y. Q. , Huang, W. D. , Yang, L. , Li, Y. T. , Lu, C. , Zhu, Y. X. , Ma, D. F. , & Yin, J. L. (2020). Genome‐wide analysis of ethylene‐insensitive3 (EIN3/EIL) in *Triticum aestivum* . Crop Science, 60, 2019–2037. 10.1002/csc2.20115

[tpg220246-bib-0014] Huang, S. , Sirikhachornkit, A. , Su, X. , Faris, J. , Gill, B. , Haselkorn, R. , & Gornicki, P. (2002). Genes encoding plastid acetyl‐CoA carboxylase and 3‐phosphoglycerate kinase of the *Triticum*/*Aegilops* complex and the evolutionary history of polyploid wheat. Proceedings of the National Academy of Sciences, 99, 8133–8138. 10.1073/pnas.072223799 PMC123033 12060759

[tpg220246-bib-0015] Huang, W. D. , He, Y. Q. , Yang, L. , Lu, C. , Zhu, Y. X. , Sun, C. , Ma, D. F. , & Yin, J. L. (2021). Genome‐wide analysis of growth‐regulating factors (GRFs) in *Triticum aestivum* . PeerJ, 9, e10701. 10.7717/peerj.10701 33552727 PMC7821759

[tpg220246-bib-0016] Jiang, W. Q. , Geng, Y. P. , Liu, Y. K. , Chen, S. H. , Cao, S. L. , Li, W. , Chen, H. G. , Ma, D. F. , & Yin, J. L. (2020). Genome‐wide identification and characterization of SRO gene family in wheat: Molecular evolution and expression profiles during different stresses. Plant Physiology and Biochemistry, 154, 590–611. 10.1016/j.plaphy.2020.07.006 32912491

[tpg220246-bib-0017] Jiang, W. Q. , Yang, L. , He, Y. Q. , Zhang, H. T. , Li, W. , Chen, H. G. , Ma, D. F. , & Yin, J. L. (2019). Genome‐wide identification and transcriptional expression analysis of superoxide dismutase (SOD) family in wheat (*Triticum aestivum*). PeerJ, 7, e8062. 10.7717/peerj.8062 31763072 PMC6873880

[tpg220246-bib-0018] Jiang, X. , Yin, J. , Wang, L. , Xi, K. , Zhu, X. , Li, G. , Zhu, Y. , & Liu, Y. (2021). Identification and evolutionary analysis of the metal‐tolerance protein family in eight Cucurbitaceae species. The Plant Genome, 15, e20167. 10.1002/tpg2.20167 34741493 PMC12807381

[tpg220246-bib-0019] June‐Sik, K. , Junya, M. , Takuya, Y. , Yasunari, F. , Jun, N. , Teppei, O. , Daisuke, T. , Kazuo, N. , Takashi, H. , Kazuo, S. , & Kazuko, Y.‐S. (2011). An ABRE promoter sequence is involved in osmotic stress‐responsive expression of the *DREB2A* gene, which encodes a transcription factor regulating drought‐inducible genes in *Arabidopsis* . Plant & Cell Physiology, 52, 2136–2146. 10.1093/pcp/pcr143 22025559

[tpg220246-bib-0020] Kawaoka, A. , & Ebinuma, H. (2001). Transcriptional control of lignin biosynthesis by tobacco LIM protein. Phytochemistry, 57, 1149–1157. 10.1016/S0031-9422(01)00054-1 11430987

[tpg220246-bib-0021] Khadiza, K. , Arif, H. K. R. , Jong‐In, P. , Nasar, U. A. , Chang, K. K. , Ki‐Byung, L. , Min‐Bae, K. , Do‐Jin, L. , Ill, S. N. , & Mi‐Young, C. (2016). Genome‐wide identification, characterization and expression profiling of *LIM* family genes in *Solanum lycopersicum* L. Plant Physiology and Biochemistry, 108, 177–190. 10.1016/j.plaphy.2016.07.006 27439220

[tpg220246-bib-0022] Kondrashov, F. A. (2012). Gene duplication as a mechanism of genomic adaptation to a changing environment. Proceedings of the Royal Society B, 279, 5048–5057. 10.1098/rspb.2012.1108 22977152 PMC3497230

[tpg220246-bib-0023] Liu, Q. , Zhang, G. Y. , & Chen, S. Y. (2001). Structure and regulatory function of plant transcription factors. Chinese Science Bulletin, 46, 271–278. 10.1007/BF02900589

[tpg220246-bib-0024] Liu, W. , Xie, Y. , Ma, J. , Luo, X. , Peng, N. , Zuo, Z. , Urs, L. , Qi, Z. , Zheng, Y. , & Zhao, Y. (2015). IBS: An illustrator for the presentation and visualization of biological sequences. Bioinformatics, 31, 3359–3361. 10.1093/bioinformatics/btv362 26069263 PMC4595897

[tpg220246-bib-0025] Maul, R. S. , Song, Y. H. , Amann, K. J. , Gerbin, S. C. , Pollard, T. D. , & Chang, D. D. (2003). EPLIN regulates actin dynamics by cross‐linking and stabilizing filaments. Journal of Cell Biology, 160, 399–407. 10.1083/jcb.200212057 12566430 PMC2172667

[tpg220246-bib-0026] Mukesh, J. , Aashima, N. , Rita, A. , Pinky, A. , Swatismita, R. , Pooja, S. , Sanjay, K. , Akhilesh, K. T. , & Jitendra, P. K. (2007). F‐box proteins in rice. Genome‐wide analysis, classification, temporal and spatial gene expression during panicle and seed development, and regulation by light and abiotic stress. Plant Physiology, 143, 1467–1483. 10.1104/pp.106.091900 17293439 PMC1851844

[tpg220246-bib-0027] Mundel, C. , Baltz, R. , Eliasson, S. , Bronner, R. , Grass, N. , Kruter, R. , Evrard, J. L. , & Steinmetz, A. (2000). A LIM‐domain protein from sunflower is localized to the cytoplasm and/or nucleus in a wide variety of tissues and is associated with the phragmoplast in dividing cells. Plant Molecular Biology, 42, 291–302. 10.1023/A:1006333611189 10794529

[tpg220246-bib-0028] Peng, J. H. , Sun, D. , & Nevo, E. (2011). Domestication evolution, genetics and genomics in wheat. Molecular Breeding, 28, 281. 10.1007/s11032-011-9608-4

[tpg220246-bib-0029] Shiu, S. H. , & Bleecker, A. B. (2003). Expansion of the Receptor‐Like Kinase/Pelle gene family and receptor‐like proteins in Arabidopsis. Plant Physiology, 132, 530–543. 10.1104/pp.103.021964 12805585 PMC166995

[tpg220246-bib-0030] Song, J. H. , Ma, D. F. , Yin, J. L. , Yang, L. , He, Y. Q. , Zhu, Z. W. , Tong, H. W. , Chen, L. , Zhu, G. , Liu, Y. K. , & Gao, C. B. (2019). Genome‐wide characterization and expression profiling of squamosa promoter binding Protein‐like (SBP) transcription factors in wheat (*Triticum aestivum* L.). Agronomy, 9, 527. 10.3390/agronomy9090527

[tpg220246-bib-0031] Song, M. , & Peng, X. (2019). Genome‐wide identification and characterization of DIR genes in *Medicago truncatula* . Biochemical Genetics, 57, 487–506. 10.1007/s10528-019-09903-7 30649641

[tpg220246-bib-0032] Thaler, J. P. , Lee, S. K. , Jurata, L. W. , Gill, G. N. , & Pfaff, S. L. (2002). LIM factor Lhx3 contributes to the specification of motor neuron and interneuron identity through cell‐type‐specific protein‐protein interactions. Cell, 110, 237–249. 10.1016/S0092-8674(02)00823-1 12150931

[tpg220246-bib-0033] Thompson, J. D. , Higgins, D. G. , & Gibson, T. J. (1994). CLUSTAL W: Improving the sensitivity of progressive multiple sequence alignment through sequence weighting, position‐specific gap penalties and weight matrix choice. Nucleic Acids Research, 22, 4673–4680. 10.1093/nar/22.22.4673 7984417 PMC308517

[tpg220246-bib-0034] Trapnell, C. R. A. , Goff, L. , Pertea, G. , Kim, D. , & Kelley, D. R. (2012). Differential gene and transcript expression analysis of RNA‐seq experiments with TopHat and Cufflinks. Nature Protocols, 7, 562–578. 10.1038/nprot.2012.016 22383036 PMC3334321

[tpg220246-bib-0035] Wang, C. , Zhang, L. J. , & Huang, R. D. (2011). Cytoskeleton and plant salt stress tolerance. Plant Signaling & Behavior, 6, 29–31. 10.4161/psb.6.1.14202 21301221 PMC3122001

[tpg220246-bib-0036] Wang, Y. , Wang, C. , Rajaofera, M. , Zhu, L. , & Miao, W. (2020). WY7 is a newly identified promoter from the rubber powdery mildew pathogen that regulates exogenous gene expression in both monocots and dicots. PLoS One, 15, e0233911. 10.1371/journal.pone.0233911 32479550 PMC7263610

[tpg220246-bib-0037] Way, J. C. , & Chalfie, M. (1988). Mec‐3, a homeobox‐containing gene that specifies differentiation of the touch receptor neurons in *C. elegans* . Cell, 54, 5–16. 10.1016/0092-8674(88)90174-2 2898300

[tpg220246-bib-0038] Yang, X. M. , Ma, J. , Li, H. B. , Ma, H. X. , Yao, J. B. , & Liu, C. J. (2010). Different genes can be responsible for crown rot resistance at different developmental stages of wheat and barley. European Journal of Plant Pathology, 128, 495–502. 10.1007/s10658-010-9680-3

[tpg220246-bib-0039] Yin, J. L. , Gu, B. , Huang, G. Y. , Tian, Y. , Quan, J. L. , Lindqvist‐Kreuze, H. , & Shan, W. X. (2017). Conserved RXLR effector genes of *Phytophthora infestans* expressed at the early stage of potato infection are suppressive to host defense. Frontiers in Plant Science, 8, 2155. 10.3389/fpls.2017.02155 29312401 PMC5742156

[tpg220246-bib-0040] Yin, J. L. , Hou, L. , Jiang, X. C. , Yang, J. , He, Y. , Zhou, X. K. , Zhu, X. M. , Gong, A. D. , Zhu, Y. X. , & Chen, Z. Y. (2021). Identification and validation of reference genes for quantitative real‐time PCR studies in alligatorweed (*Alternanthera philoxeroides*). Weed Science, 69, 404–411. 10.1017/wsc.2021.32

[tpg220246-bib-0041] Yin, J. L. , Liu, M. Y. , Ma, D. F. , Wu, J. W. , Li, S. L. , Zhu, Y. X. , & Han, B. (2018). Identification of circular RNAs and their targets during tomato fruit ripening. Postharvest Biology and Technology, 136, 90–98. 10.1016/j.postharvbio.2017.10.013

[tpg220246-bib-0042] Yin, J. L. , Wang, L. X. , Zhao, J. , Li, Y. T. , Huang, R. , Jiang, X. C. , Zhou, X. K. , Zhu, X. M. , He, Y. , He, Y. Q. , Liu, Y. Q. , & Zhu, Y. X. (2020). Genome‐wide characterization of the C2H2 zinc‐finger genes in *Cucumis sativus* and functional analyses of four *CsZFPs* in response to stresses. BMC Plant Biology, 20, 359. 10.1186/s12870-020-02575-1 32727369 PMC7392682

[tpg220246-bib-0043] Zhang, H. Y. , Li, Z. T. , Zhao, C. J. , Yang, K. J. , Wang, Y. F. , Hu, X. W. , & Zhao, Y. (2013). Genome‐wide analysis of LIM domain‐containing protein gene family in Maize. Journal of Maize Sciences, 21, 40–47. 10.13597/j.cnki.maize.science.2013.03.010

[tpg220246-bib-0044] Zhang, P. G. , Zhu, Y. X. , Ma, D. F. , Xu, W. J. , Zhou, J. J. , Yan, H. W. , Yang, L. , & Yin, J. L. (2019). Screening, identification, and optimization of fermentation conditions of an antagonistic endophyte to wheat head blight. Agronomy, 9, 476. 10.3390/agronomy9090476

[tpg220246-bib-0045] Zhou, R. , Zhu, Y. X. , Zhao, J. , Fang, Z. W. , Wang, S. P. , Yin, J. L. , Chu, Z. H. , & Ma, D. F. (2018). Transcriptome‐wide identification and characterization of potato circular RNAs in response to *Pectobacterium carotovorum* Subspecies *brasiliense* infection. International Journal of Molecular Sciences, 19, 71. 10.3390/ijms19010071 PMC5796021 29280973

[tpg220246-bib-0046] Zhou, X. Y. , Zhu, X. G. , Shao, W. N. , Song, J. H. , Jiang, W. Q. , He, Y. Q. , Yin, J. L. , Ma, D. F. , & Qiao, Y. L. (2020). Genome‐wide mining of wheat *DUF966* gene family provides new insights into salt stress responses. Frontiers in Plant Science, 11, 569838. 10.3389/fpls.2020.569838 32983219 PMC7483657

[tpg220246-bib-0047] Zhu, Y. X. , Gong, H. J. , & Yin, J. L. (2019). Role of silicon in mediating salt tolerance in plants: A review. Plants, 8, 147. 10.3390/plants8060147 31159197 PMC6630593

[tpg220246-bib-0048] Zhu, Y. X. , Jia, J. H. , Yang, L. , Xia, Y. C. , Zhang, H. L. , Jia, J. B. , Zhou, R. , Nie, P. Y. , Yin, J. L. , Ma, D. F. , & Liu, L. C. (2019). Identification of cucumber circular RNAs responsive to salt stress. BMC Plant Biology, 19, 164. 10.1186/s12870-019-1712-3 31029105 PMC6486992

[tpg220246-bib-0049] Zhu, Y. X. , Jiang, X. C. , Han, X. W. , Han, S. , Chen, Z. Y. , Yin, J. L. , & Liu, Y. Q. (2021). Characterization the coding and non‐coding RNA components in the transcriptome of invasion weed *Alternanthera philoxeroides* . Notulae Botanicae Horti Agrobotanici Cluj‐Napoca, 49, 12242. 10.15835/nbha49112242

[tpg220246-bib-0050] Zhu, Y. X. , Jiang, X. C. , Zhang, J. , He, Y. , Zhu, X. M. , Zhou, X. K. , Gong, H. J. , Yin, J. L. , & Liu, Y. Q. (2020). Silicon confers cucumber resistance to salinity stress through regulation of proline and cytokinins. Plant Physiology and Biochemistry, 156, 209–220. 10.1016/j.plaphy.2020.09.014 32977177

[tpg220246-bib-0051] Zhu, Y. , Wu, N. N. , Song, W. L. , Yin, G. J. , Qin, Y. J. , Yan, Y. M. , & Hu, Y. K. (2014). Soybean (*Glycine max*) expansin gene superfamily origins: Segmental and tandem duplication events followed by divergent selection among subfamilies. BMC Plant Biology, 14, 93. 10.1186/1471-2229-14-93 24720629 PMC4021193

[tpg220246-bib-0052] Zhu, Y. X. , Yang, L. , Liu, N. , Yang, J. , Zhou, X. K. , Xia, Y. C. , He, Y. , He, Y. Q. , Gong, H. J. , Ma, D. F. , & Yin, J. L. (2019). Genome‐wide identification, structure characterization, and expression pattern profiling of aquaporin gene family in cucumber. BMC Plant Biology, 19, 345. 10.1186/s12870-019-1953-1 31390991 PMC6686268

