# Supplementary material for: Genome‐wide characterization and function analysis uncovered roles of wheat LIMs in responding to adverse stresses and TaLIM8‐4D function as a susceptible gene
Source: Plant Genome. 2022 Jul 27;15(3):e20246. doi: 10.1002/tpg2.20246 (PMC12807053; doi:10.1002/tpg2.20246)
Supplement: Supplementary file 1 [file TPG2-15-e20246-s001.docx]

Supplementary Figure 1. Chromosomal distribution of *LIMs* of *Triticum aestivum* (Ta), *Triticum urartu* (Tu), *Triticum dicoccoides* (Td), and *Aegilops tauschii* (Ae). The ruler on the left represents the physical map distance among genes (Mbp).
